# Supplementary material for: The Toll-Like Receptor 5 agonist flagellin prevents Non-typeable Haemophilus influenzae-induced infection in cigarette smoke-exposed mice
Source: PLoS One. 2021 Mar 30;16(3):e0236216. doi: 10.1371/journal.pone.0236216 (PMC8009382; doi:10.1371/journal.pone.0236216)
Supplement: S1 Fig — (PDF) [file pone.0236216.s001.pdf]

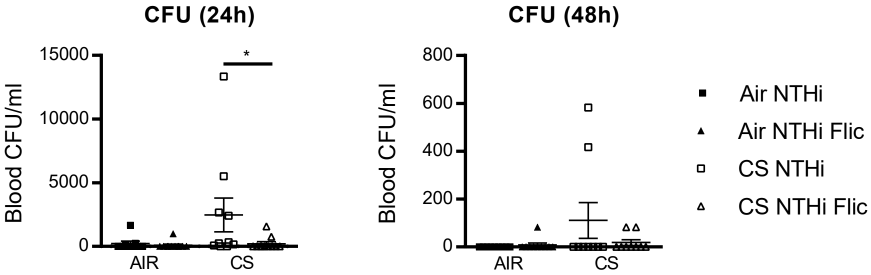

Supplementary figure 1. Treatment with Flagellin prevents the blood dissemination of NTH in CS-exposed mice. (a) Colony Forming Unit (CFU) counts in Blood. Three independent experiments have been performed with 4 mice in each group. Data are expressed as mean ± SEM. *: $p<0.05$.
