# Supplementary material for: The Toll-Like Receptor 5 agonist flagellin prevents Non-typeable Haemophilus influenzae-induced infection in cigarette smoke-exposed mice
Source: PLoS One. 2021 Mar 30;16(3):e0236216. doi: 10.1371/journal.pone.0236216 (PMC8009382; doi:10.1371/journal.pone.0236216)
Supplement: S2 Fig — (PDF) [file pone.0236216.s002.pdf]

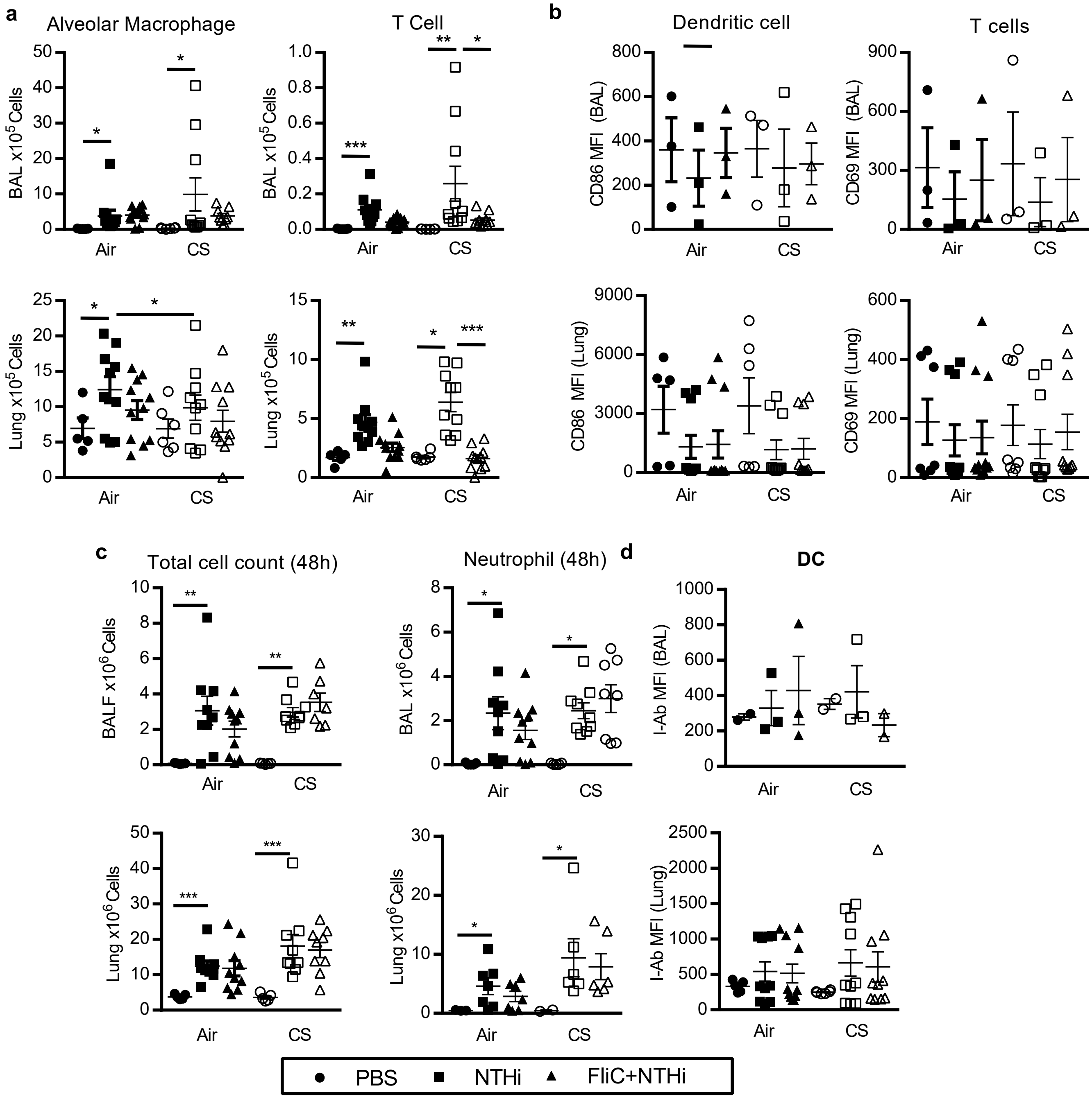


**Supplementary figure 2: Flagellin modulated the inflammatory cell recruitment whereas it did not affect their activation in NTHi-infected cigarette smoke-exposed mice.**
(a) Absolute numbers of alveolar macrophages and T cells were defined in the BAL and the lungs of mice infected or not with NTHi and treated or not with FliC, at 24h after infection. (b) Activation of dendritic cells and T cells at 24h post-infection were evaluated by the expression of the MHC molecules I-Ab and CD69, respectively, in both the BAL and the lungs cells. (c) Total cell and neutrophil numbers were determined in the BAL and the lungs of mice infected or not with NTHi and treated or not with FliC, at 48h after infection. (b) Activation of dendritic cells at 48h post-infection were evaluated by the expression of I-Ab, in both the BAL and the lungs cells. Cell activation was reported as median fluorescence intensity (MFI). Three independent experiments have been performed with at least 3 mice in each group. Results were obtained by flow cytometry and the data are expressed as mean ± SEM. *: $p<0.05$, **: $p<0.01$, ***: $p<0.001$.
