# Supplementary material for: The Toll-Like Receptor 5 agonist flagellin prevents Non-typeable Haemophilus influenzae-induced infection in cigarette smoke-exposed mice
Source: PLoS One. 2021 Mar 30;16(3):e0236216. doi: 10.1371/journal.pone.0236216 (PMC8009382; doi:10.1371/journal.pone.0236216)
Supplement: S3 Fig — (PDF) [file pone.0236216.s003.pdf]

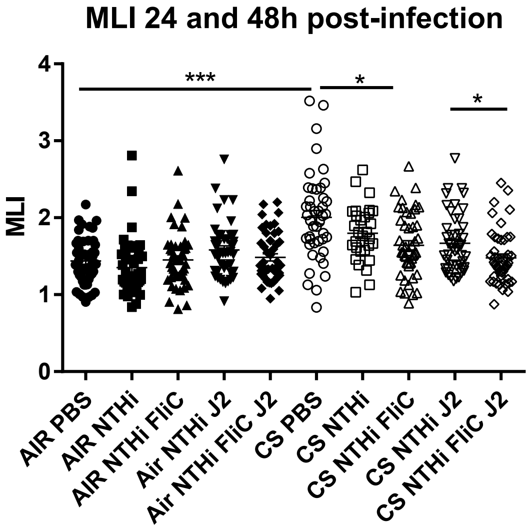

Supplementary figure 3: Flagellin reduce the emphysema in the lung of NTHi-infected cigarrette smoke-exposed mice. Emphysema was reported as median mean linear intercept (MLI). Three independent experiments have been performed with at least 3 mice in each group. Results were obtained by Image sofware and the data are expressed as mean ± SEM. *: p<0.05, ***: p<0.001.
