# Supplementary material for: The Toll-Like Receptor 5 agonist flagellin prevents Non-typeable Haemophilus influenzae-induced infection in cigarette smoke-exposed mice
Source: PLoS One. 2021 Mar 30;16(3):e0236216. doi: 10.1371/journal.pone.0236216 (PMC8009382; doi:10.1371/journal.pone.0236216)
Supplement: S4 Fig — (PDF) [file pone.0236216.s004.pdf]

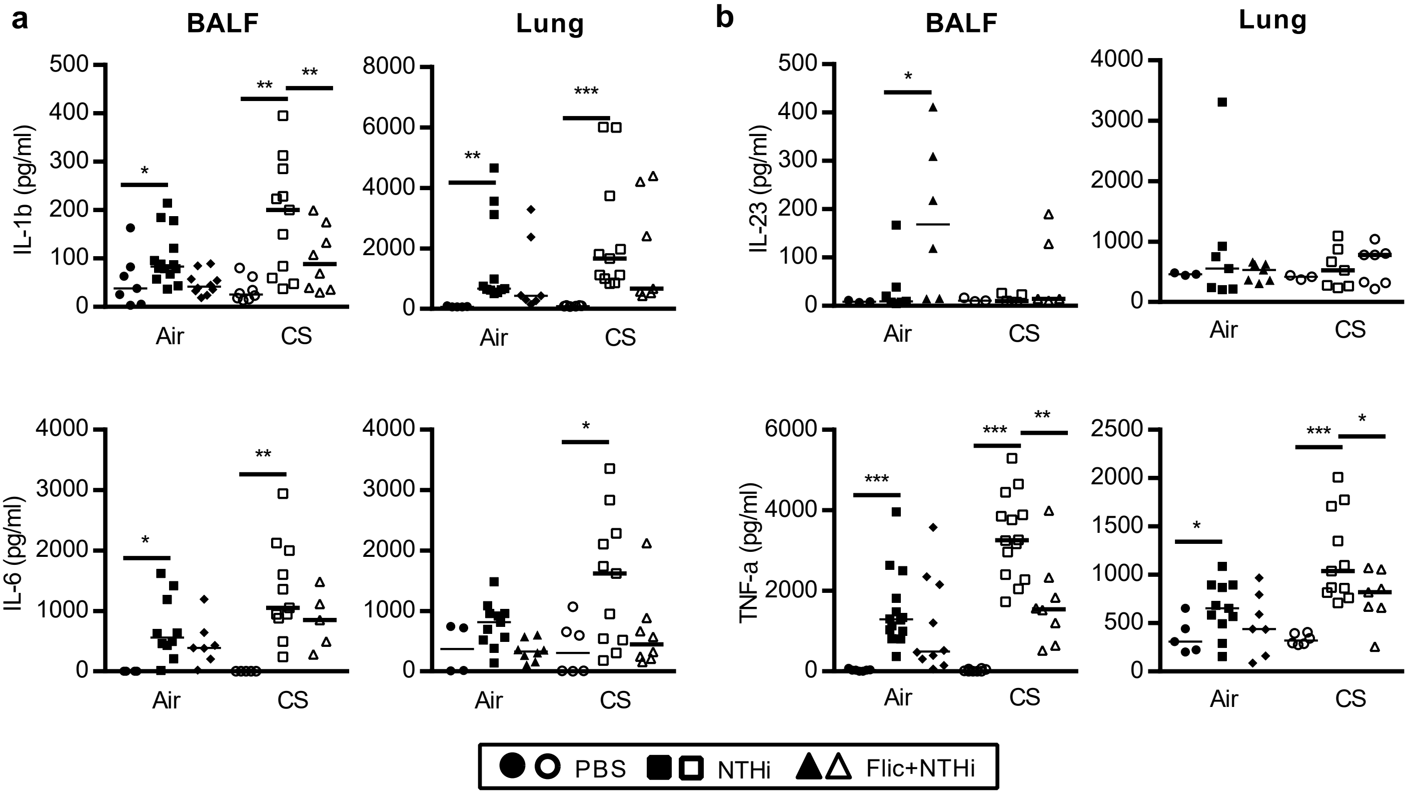

Supplementary figure 4: Modulation by flagellin of cytokine production in the lung of Air- and cigarette smoke-exposed mice infected with NTHi. Concentrations of inflammatory cytokines ((a) IL-1b, IL-6); (b) TNF-a and IL-23) were measured in the BAL fluid and the lungs of control and cigarette smoke-exposed mice infected (24h) or not with NTHi and treated or not with flagellin (FliC). Cytokine concentrations were determined by ELISA. Three independent experiments have been performed with 4 mice in each group. The data are expressed as mean ± SEM. *: $p<0.05$, **: $p<0.01$, ***: $p<0.001$.
