# Supplementary material for: The Toll-Like Receptor 5 agonist flagellin prevents Non-typeable Haemophilus influenzae-induced infection in cigarette smoke-exposed mice
Source: PLoS One. 2021 Mar 30;16(3):e0236216. doi: 10.1371/journal.pone.0236216 (PMC8009382; doi:10.1371/journal.pone.0236216)
Supplement: S5 Fig — (PDF) [file pone.0236216.s005.pdf]

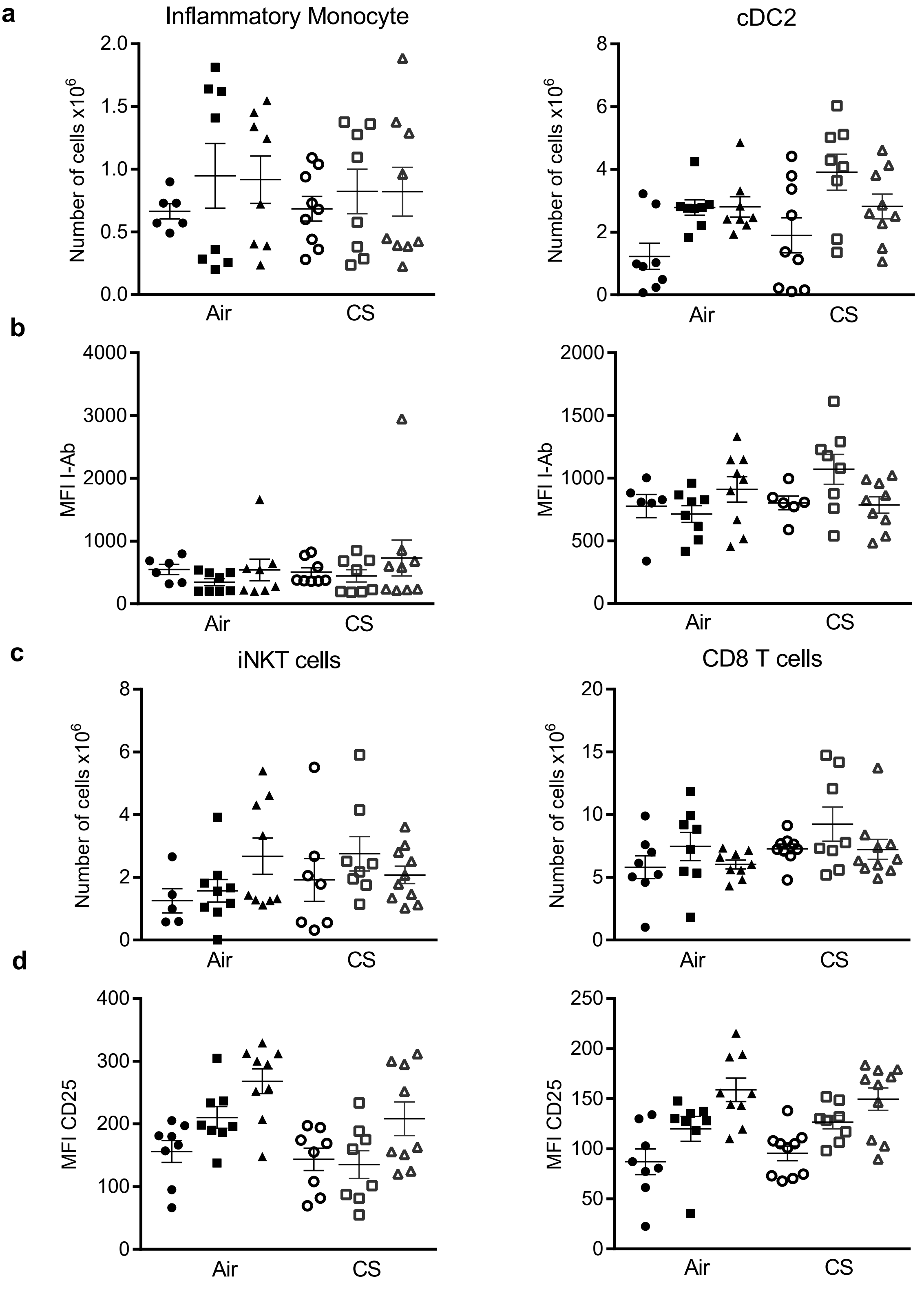

Supplemental figure 5: Flagellin did not affect the main immune cell populations nor their activation in the spleen of NTHi-infected cigarette smoke-exposed mice.
(a) Absolute numbers of inflammatory monocytes and conventionnal dendritic cells 2 (cDC2) were defined in the spleen of mice infected or not with NTHi and treated or not with FliC, at 48h after infection. (b) Activation of antigen-presenting cells was evaluated by the expression of the MHC molecules I-Ab. (c) Absolute numbers of invariant natural killer cells (iNKT) and $CD8^+$ T lymphocytes were defined in the spleen of mice infected or not with NTHi and treated or not with FliC, at 48h after infection. (d) Activation of lymphocytes was evaluated by the expression of CD25 in spleen cells. Cell activation was reported as median fluorescence intensity (MFI). Three independent experiments have been performed with at least 3 mice in each group. There was no statistical difference.
