# Supplementary material for: The Toll-Like Receptor 5 agonist flagellin prevents Non-typeable Haemophilus influenzae-induced infection in cigarette smoke-exposed mice
Source: PLoS One. 2021 Mar 30;16(3):e0236216. doi: 10.1371/journal.pone.0236216 (PMC8009382; doi:10.1371/journal.pone.0236216)
Supplement: S6 Fig — (PDF) [file pone.0236216.s006.pdf]

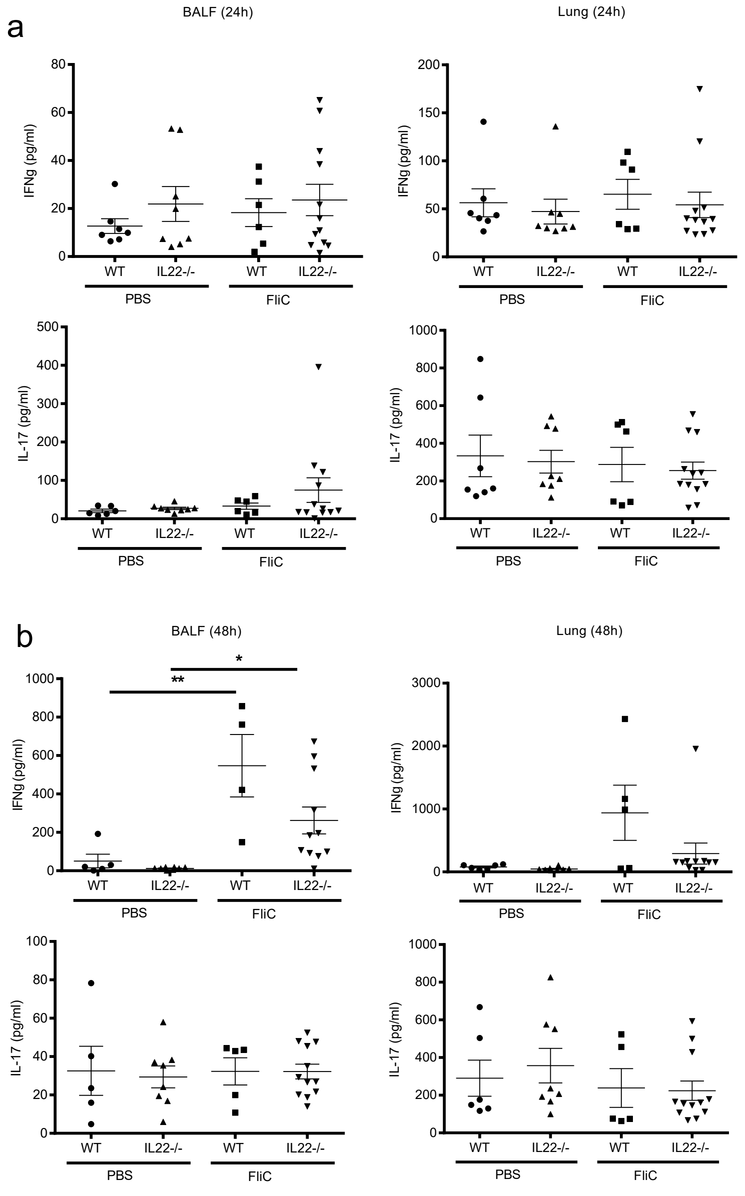

Supplementary figure 6: Cytokine production in the lung of WT and IL-22$^{-/-}$ mice following flagellin treatment. Concentrations of inflammatory cytokines (IL-17 and IFN-g) were measured in the BAL fluid and the lungs of control and IL-22$^{-/-}$ mice treated or not with FliC at 24h (a) and 48h (b) post infection by NTHi. Cytokine concentrations were determined by ELISA. Three independent experiments have been performed with at least 4 mice in each group. The data are expressed as mean ± SEM. *: $p<0.05$, **: $p<0.01$.
