# Supplementary material for: The Toll-Like Receptor 5 agonist flagellin prevents Non-typeable Haemophilus influenzae-induced infection in cigarette smoke-exposed mice
Source: PLoS One. 2021 Mar 30;16(3):e0236216. doi: 10.1371/journal.pone.0236216 (PMC8009382; doi:10.1371/journal.pone.0236216)
Supplement: S7 Fig — (PDF) [file pone.0236216.s007.pdf]

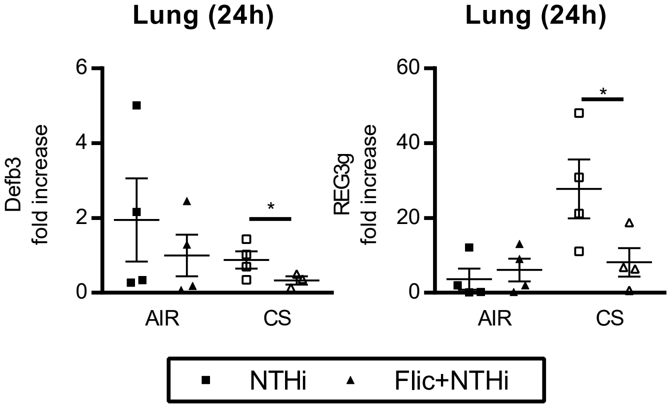

Supplementary figure 7 : Flagellin reduce the Defb3 and REG3g mRNA expression in the lung of NTHi-infected cigarette smoke-exposed mice. Results were obtained by qPCR and the data are expressed as mean ± SEM. *: $p<0.05$.
